# A DIC-Based Study on Fatigue Damage Evolution in Pre-Corroded Aluminum Alloy 2024-T4

**DOI:** 10.3390/ma11112243

**Published:** 2018-11-11

**Authors:** Haipeng Song, Changchun Liu, Hao Zhang, Sean B. Leen

**Affiliations:** 1 Sino-European Institute of Aviation Engineering, Civil Aviation University of China, Tianjin 300300, China; ningdulaicun@163.com; 2 Mechanical Engineering, College of Engineering and Informatics, H91 HK31 Galway, Ireland; sean.leen@nuigalway.ie; 3 College of Mechanical Engineering, Yangzhou University, Yangzhou 225127, China; zhanghaosteven@163.com

**Keywords:** aluminum alloy, prior corrosion, fatigue damage, crack growth, digital image correlation

## Abstract

This paper investigates the fatigue damage and cracking behavior of aluminum alloy 2024-T4 with different levels of prior corrosion. Damage evolution, crack initiation and propagation were experimentally analyzed by digital image correlation, scanning electron microscopy and damage curves. Prior corrosion is shown to cause accelerated damage accumulation, inducing premature fatigue crack initiation, and affecting crack nucleation location, crack orientation and fracture path. For the pre-corrosion condition, although multiple cracks were observed, only one corrosion-initiated primary crack dominates the failure process, in contrast to the plain fatigue cases, where multiple cracks propagated simultaneously leading to final coalescence and fracture. Based on the experimental observations, a mixed-mode fracture model is proposed and shown to successfully predict fatigue crack growth and failure from the single dominant localized corrosion region.

## 1. Introduction

The heterogeneous microstructure of high strength aluminum alloys (AA) such as 2xxx and 7xxx, widely used in aircraft structures, renders them highly susceptible to localized corrosion, such as pitting corrosion and exfoliation corrosion, in service environments [1,2]. The combined effect of localized corrosion and fatigue loading adversely affects aircraft structural integrity, which is considered to be one of the most significant damage mechanisms in aging aircraft [3,4]. It is therefore necessary and critical to understand the corrosion-enhanced fatigue damage and cracking behavior of aeronautic aluminum alloys, for flight safety and the economic maintenance of aircraft.

A number of studies have previously experimentally investigated the fatigue performance of aluminum alloys with localized corrosion [5,6,7,8]. AA7475-T761 samples after corrosion exposure were subjected to constant and variable amplitude fatigue loading. Pre-existing localized corrosion was shown to reduce fatigue life by 40 to 50%, compared with un-corroded samples [9]. Similarly, bending fatigue experiments on pre-corroded AA7075-T6 showed that fatigue strength was reduced by about 60% due to the presence of corrosion pits [10]. Such observed significant degradation in fatigue properties is attributable to premature crack initiation and propagation originating from localized corrosion [11,12,13,14,15]. Fractographic observations on fatigue crack formation in pre-corroded AA2024-T3 alloy under fatigue loading showed that corrosion pits caused accelerated multiple-site crack initiation [16]; elsewhere, cracks in AA7075-T6511 were shown to initiate from pits clustered as a semi-eliptical surface micro notch, rather than the deepest pits [15]. Interrupted tests conducted on pre-corroded AA2024-T3, to investigate crack initiation in corrosion-nucleated fatigue, showed that crack initiation was essentially immediate upon application of cyclic loading [17]. Cracks originating from corrosion pits were visually investigated using microscopy techniques in order to gain insight into the pit-to-crack transition process of AA2024-T3; the results indicated that quantities such as pit surface area and surrounding pit proximity are as important as pit depth in determining when and where cracks will form [18]. It has been also observed that the pit-to-crack transition depends upon the topographic and microscopic features of the corrosion pit as well as upon the applied fatigue stress level [19]. Noelle et al. applied a crack surface marker-band method to determine the short crack growth rate of AA7050-T7451 samples with macro-scale corrosion damage; crack growth rates were shown to converge to comparable values for varied corrosion morphologies [20]. The effect of prior corrosion on fatigue crack propagation in aluminum alloy 6151-T6 was investigated via in-situ scanning electron microscopy (SEM) tests; it was found that the early stage of fatigue micro crack propagation behavior can be described by K_I_/K_II_-mixed mode fracture [21]. The specimen surfaces were monitored by optical microscopy to investigate the short crack growth of pre-corroded AA7075-T6; this revealed that short crack growth life under salt water conditions was about 5 to 8 times lower than in ambient air [22].

Some modelling work has also been conducted to analyze the damage accumulation and crack growth in aluminum alloys considering the coupled effect of fatigue and corrosion, mainly based on damage mechanics and fracture mechanics. An approach based on continuum damage mechanics was applied to predict corrosion-fatigue crack initiation life of AA2024-T62, taking account the elastic damage accumulation [23]. The pit-to-crack initiation life in AA2024-T3 was studied using a damage mechanics model based on the single dominant flaw approach. It was assumed that although several pits may be present, only one pit grows to a critical depth and transitions to a fatigue crack [24]. Coupled elastic-plastic damage evolution models were adopted to predict the corrosion fatigue life of aluminum alloy [1]. In the fracture mechanics-based approach, localized corrosion regions were usually considered to be equivalent to surface cracks. A crack growth accumulative methodology based on the Willenborg-Chang rule was used to evaluate the residual life of aluminum alloy under corrosion-fatigue conditions [25]. Linear elastic fracture mechanics modelling, using a corrosion modified-equivalent initial flaw size, was adopted to predict fatigue life of field corroded AA7075-T6511 under variable amplitude loading [7]. Xiang et al. combined an asymptotic stress intensity factor (SIF) solution of model-I crack and corrosion pit growth function for fatigue life prediction of pre-corroded specimens [26].

However, there is still a lack of quantitative, full-field observation of fatigue failure to analyze the temporal and spatial characteristics of damage evolution, crack initiation and propagation in pre-corroded aluminum alloy. Moreover, the corresponding models for post-corrosion fatigue life prediction need to be further developed to consider the identified damage and cracking characteristics, such as mixed-mode fracture. Digital image correlation (DIC) is an optical method which can quantify the deformation field on the specimen surface with the advantages of providing full-field, real-time, and non-contact measurements, as well as flexibility [27,28,29,30]. The method has been successfully applied to observe damage and failure in metallic materials during fatigue tests [31,32]. Short cracks during intergranular stress-corrosion cracking of austenitic stainless steel were detected by DIC [33]. DIC was also used to monitor short fatigue crack propagation of austenitic stainless steel in oxygenated water [34]. Recently, damage evolution and crack propagation of pre-corroded AA7050-T7651 in uniaxial tension tests was observed by DIC [35]. However, to our knowledge, macroscopic fatigue damage evolution and crack propagation in aluminum alloy specimens with prior corrosion has not previously been studied by DIC. 

Considering that aircraft structures typically experience corrosion during ground parking and fatigue loading during flight [2], the fatigue damage and cracking behavior in aluminum alloy with prior corrosion is analyzed in the present work. Since S-N curves of pre-corroded aluminum alloy have already been widely discussed [2,6,10,23], we focus here on analysis of fatigue damage accumulation, crack initiation and propagation by DIC and SEM, as well as the damage and mixed-mode fracture modeling. Constant amplitude fatigue tests with typical stress levels were conducted on AA2024-T4 with different levels of initial pre-corrosion damage. The measured fatigue damage curves are described using a non-linear damage model. 3D-DIC was used to obtain the strain fields during the fatigue process, to visually display damage evolution and crack propagation in the test specimens up to failure. The fracture surfaces of key damage regions, determined by DIC, were further examined to identify crack initiation features. With the combined analysis of damage curves, strain field evolution and fracture morphology, a detailed experimental description of the entire corrosion-nucleated fatigue failure process is achieved. Based on the experimental observations, a mixed-mode fracture model is developed to simulate fatigue crack growth originated from a single dominant corrosion pit and, hence, predict residual fatigue life.

## 2. Experiment

### 2.1. Material and Specimen

**Figure 1:**
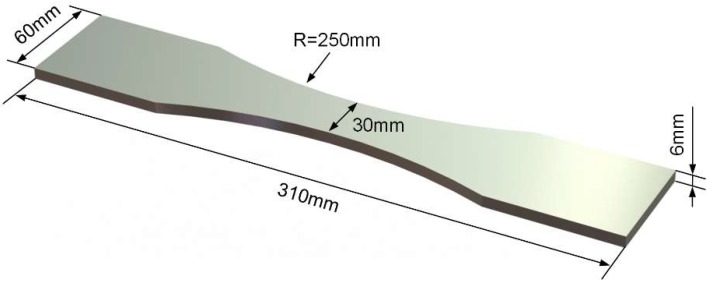
Schematic diagram of specimen for fatigue tests.

**Figure 2:**
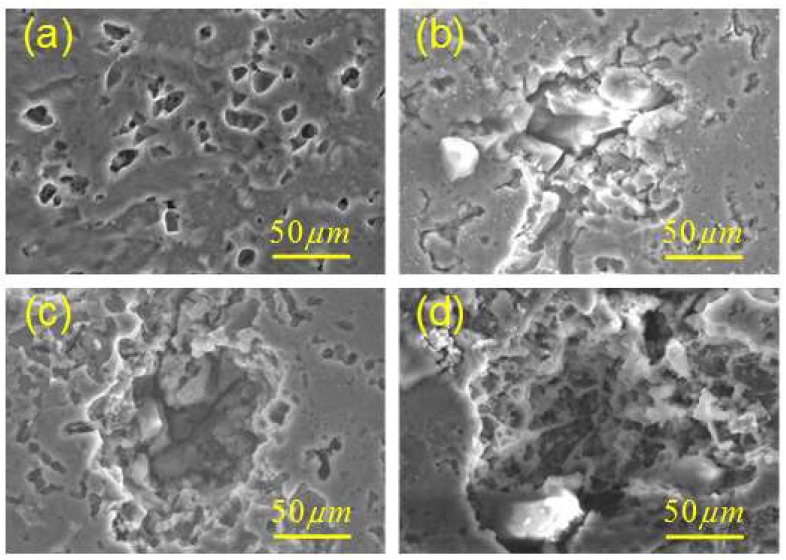
Corrosion morphology of aluminum alloy 2024-T4 exposed to EXCO solution for different times (**a**) 0 h, (**b**) 24 h, (**c**) 48 h, (**d**) 96 h.

The as-received material was in the form of a 6 mm thick, rolled aluminum alloy 2024-T4 plate (Si-0.5 Ti-0.15 Fe-0.5 Mg-1.76 Cu-3.77 Mn-0.37 Al; wt %). The monotonic tensile yield strength was measured as 352 MPa and the ultimate tensile strength as 492 MPa. Dog-bone specimens were chosen for the fatigue tests, as shown in Figure 1. Prior to fatigue, three groups of specimens (with three repeat samples in each group) were exposed to the laboratory exfoliation corrosion (EXCO) environment for 0, 48 and 96 h, respectively, to generate three different levels of initial corrosion damage. According to specification ASTM G34-01 [36], the corrosive solution consisted of the following chemicals diluted in 1 L distilled water: Sodium chloride (234 g NaCl), potassium nitrate (50 g KNO_3_) and nitric acid (6.3 mL HNO_3_). Typical corrosion morphologies of aluminum alloy 2024-T4 after exposure in EXCO resolution are shown in Figure 2. It was observed that the surface and depth of localized corrosion damage increased with increasing exposure time, indicating the severe detrimental impact of the corrosive solution on the aluminum alloy 2024-T4. After corrosion, all specimens were rinsed and ultrasonically cleaned for 15 min in ethyl alcohol, then dried. To facilitate DIC analysis, random speckle patterns were applied by spraying white and black paint on the surfaces of all specimens before loading.

### 2.2. Test System and Experimental Procedure

**Figure 3:**
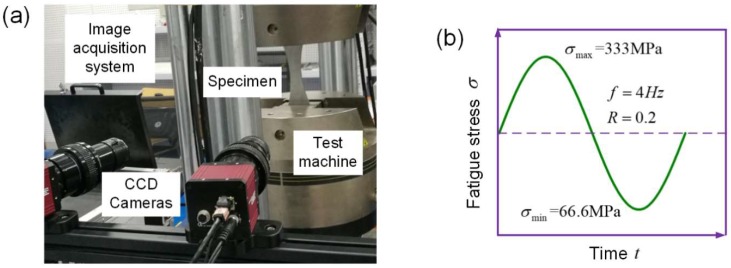
(**a**) Experimental set-up for fatigue tests on aluminum alloy, (**b**) applied fatigue stress waveform.

Constant amplitude uniaxial fatigue testing was successively performed on the three groups of specimens in laboratory air at room temperature. Three repeat tests were done, at the same loading condition, for each corrosion case. Figure 3a shows the experimental set-up, including the loading system and image acquisition system. Specimens were loaded in a closed loop servo-hydraulic Instron-8803 fatigue test machine with a loading capacity of 250 kN. The applied axial load was controlled to apply a sine wave load in all the tests. As shown in Figure 3b, the maximum applied-uniform tensile stress was 333 MPa, with a stress ratio (R) of 0.2, and frequency of 4 Hz. 

During the fatigue process, stereo images of the specimen surface at zero load level at specified fatigue cycles were captured by the commercial software VIC-3D (Version 7, Correlated Solutions, Inc., Irmo, SC, USA). Since the quality of captured speckle images in active status is lower than that in the static condition, the load was temporarily interrupted to maintain zero when taking the images. The resolution of the CCD capture region was 1524 × 3205 pixels. The DIC system was calibrated using a calibration plate to obtain the camera parameters. Then, the acquired stereo image sequences of specimens were processed by VIC-3D software, which determined the three-dimensional positions before and after deformation, by tracking the grey value pattern in small subsets throughout the image sequence. Here, the subset size was chosen as 27 × 27 pixels, and the step length was 5 pixels. The displacements measured in the analysis region were then numerically differentiated to compute the surface strain fields using the VIC-3D software.

## 3. Results

### 3.1. Fatigue Lives

**Figure 4:**
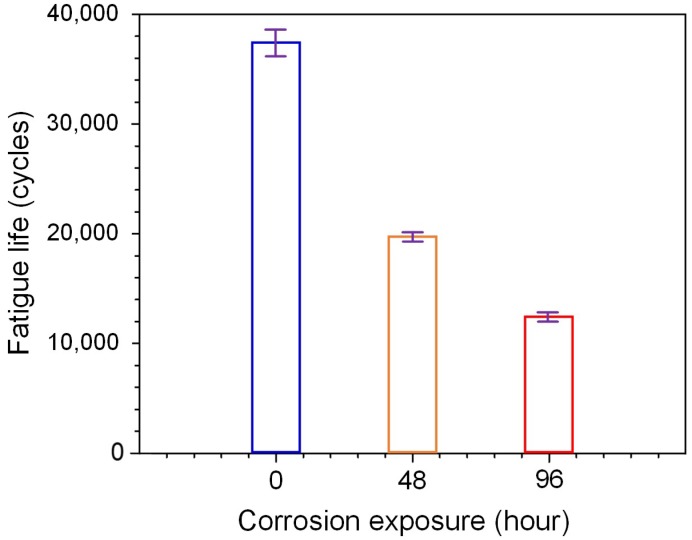
Measured effect of corrosion time on fatigue life at a stress level of σ_max_ = 330 MPa, *R* = 0.2.

Figure 4 shows the fatigue lives of specimens with different levels of localized corrosion damage. The average numbers of cycles to failure were 3.75 × 10^4^, 1.98 × 10^4^ and 1.20 × 10^4^, corresponding to the specimens with corrosion times of 0, 48 and 96 h respectively. The fatigue life thus reduced by about 47.2% after 48 h exposure compared to that without corrosion, and reduced by about 68% after 96 h exposure. These experimental results clearly show that the presence of localized corrosion has a detrimental effect on fatigue performance of aluminum alloy 2024-T4.

### 3.2. Fatigue Damage Accumulation

Based on the concept of continuum damage mechanics [37], a fatigue-induced damage variable is defined to represent the nucleation and growth of microviods and microcracks in the material, calculated by the degradation of elastic modulus:

(1) $$D_{f}=1-\frac{\tilde{E}}{E}\;$$

where E is the initial elastic modulus of the material and $\tilde{E}$ is the elastic modulus of the damaged material affected by fatigue loading.

In the case of assumed isotropic damage, fatigue-induced damage in stress-controlled fatigue tests can be approximately obtained based on the strain evolution during the fatigue process:

(2) $$D_{f}(N)=1-\frac{\varepsilon (1)}{\varepsilon \left(N\right)}\;$$

where $D_{f}(N)$ is the fatigue damage for cycle N in a given stress controlled fatigue test, ε(1) is the maximum tensile strain for the first cycle, and ε(N) is the maximum tensile strain for cycle N in the stress controlled fatigue test. Thus, fatigue damage $D_{f}$ equals to 0 in the first cycle; and reaches the maximum value in the final cycle.

**Figure 5:**
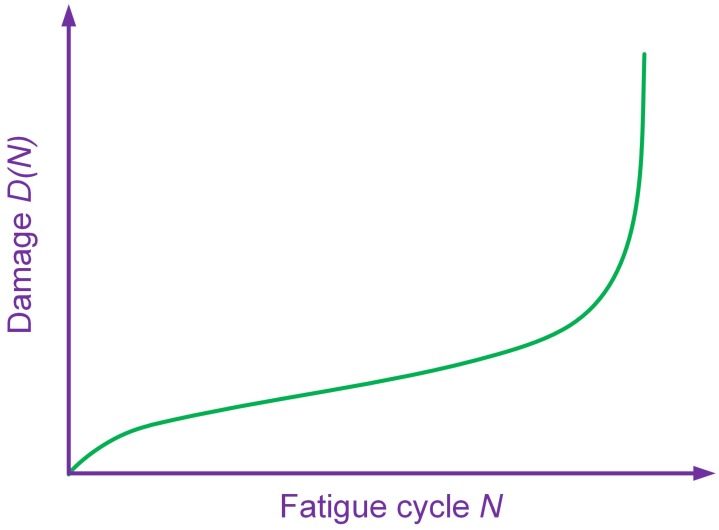
Schematic diagram of inverted S-shaped fatigue damage model.

Corresponding to the experimental data, an inverted S-shaped fatigue damage model [38], as shown in Figure 5, is used to describe the fatigue damage evolution, as a function of number of fatigue cycles N:

(3) $$D_{f}(N)=\alpha {\left[\frac{\beta }{\beta -\frac{N}{N_{f}}}-1\right]}^{1/p}\;$$

where α, β and *p* are damage parameters, which can be identified by fitting the modelling results to experimental data. Here, the fitting process was conducted via a Matlab built-in function ‘nlinfit’ based on least squares estimation. The identified damage parameters for specimens with different corrosion times are shown in Table 1.

**Figure 6:**
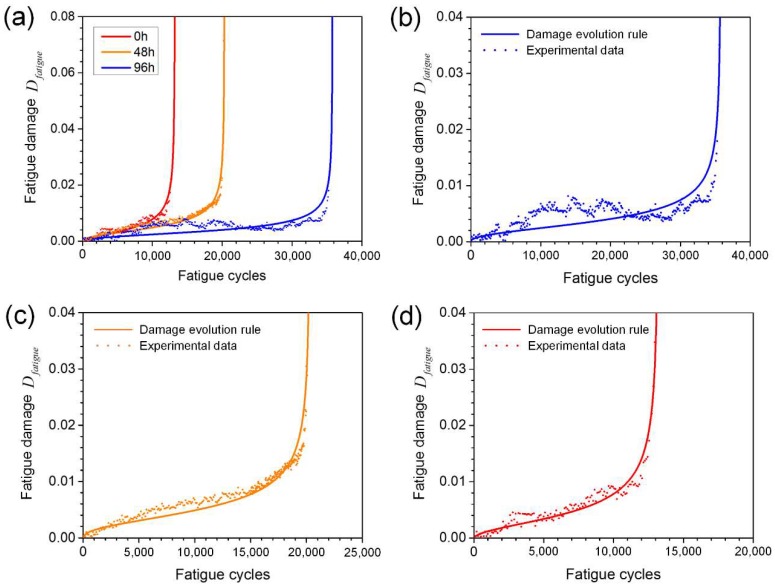
Measured and fitted damage curves during fatigue tests for specimens with different corrosion times (**a**) 0, 48 and 96 h, (**b**) 0 h, (**c**) 48 h, (**d**) 96 h.

Figure 6 shows the experimental and fitting damage curves as a function of fatigue cycles for the specimens with corrosion times of 0, 48 and 96 h, based on Equation (2) and Equation (3), reflecting the evolution of fatigue induced damage in specimens with different levels of initial corrosion damage. A small increase in the fatigue damage can be observed in all curves during the early stage of fatigue; then, after an inflection point, a more rapid increase of fatigue damage occurs leading to rupture. This response is attributed to the fast propagation of a primary crack. It is seen that prior corrosion leads to significantly accelerated fatigue damage accumulation, and hence reducing life, with increasing corrosion time.

### 3.3. Crack Initiation and Propagation by 3D-DIC

**Figure 7:**
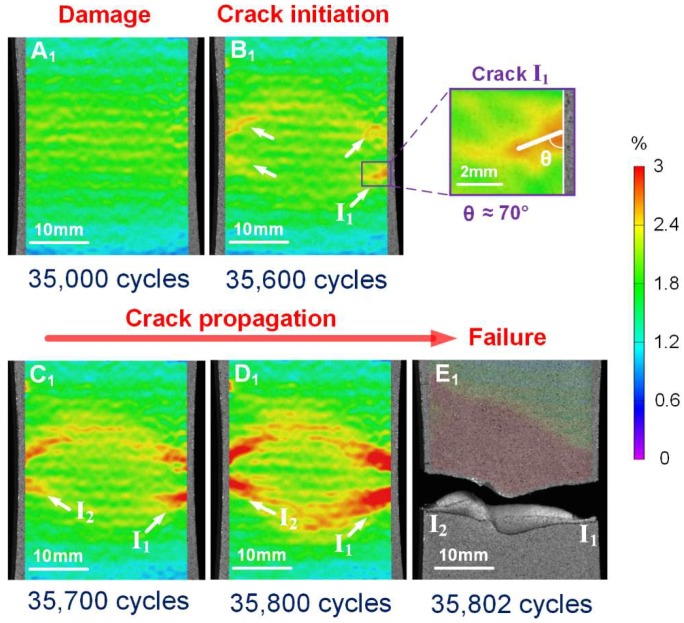
Measured contour-plot distribution of evolution of maximum tensile strain, facilitating visualization of damage, crack initiation and propagation for corrosion time of 0 h.

The maximum tensile strain fields on the specimen surfaces at specified fatigue cycles were calculated by 3D-DIC to reveal the details of macro-crack initiation and propagation in specimens with different corrosion times. Figure 7 illustrates typical DIC analysis results in an un-corroded specimen, corresponding to zero load level in the denoted fatigue cycles. No evident damage localization was detected before 3.5 × 10^4^ cycles (A_1_ in Figure 7). Some slight horizontal bands appeared on the specimen surface may be attributed to effects of the rolling process. As the cycles increased to 3.56 × 10^4^, four separated strain localization regions appeared at the edges of the specimen (B_1_ in Figure 7, indicated by the white arrow), reflecting gradual localization of damage, leading to crack initiation. From the enlarged view of key region I_1_, a crack was detected with orientation $\theta \approx 70^{\circ }$ from the vertical direction. Considering that crack closure effects would have some adverse influences on fatigue crack length measurement [39], here we just identify the crack initiation and orientation by DIC; a deeper analysis on crack length and crack closure will be conducted via small-scale DIC in future work. It seems reasonable that these crack initiation regions were located near the mid-length of the specimen, since the section area here is the smallest according to the sample size (Figure 1). The cracks in each region propagated simultaneously with increasing numbers of fatigue cycles (B_1_, C_1_ and D_1_ in Figure 7). The strain fields show that the lengths and widths of the cracks became larger and larger. Finally, the coalescence of two main cracks, originated from region I_1_ and I_2_, resulted in catastrophic failure at 3.58 × 10^4^ cycles (E_1_ in Figure 7).

**Figure 8:**
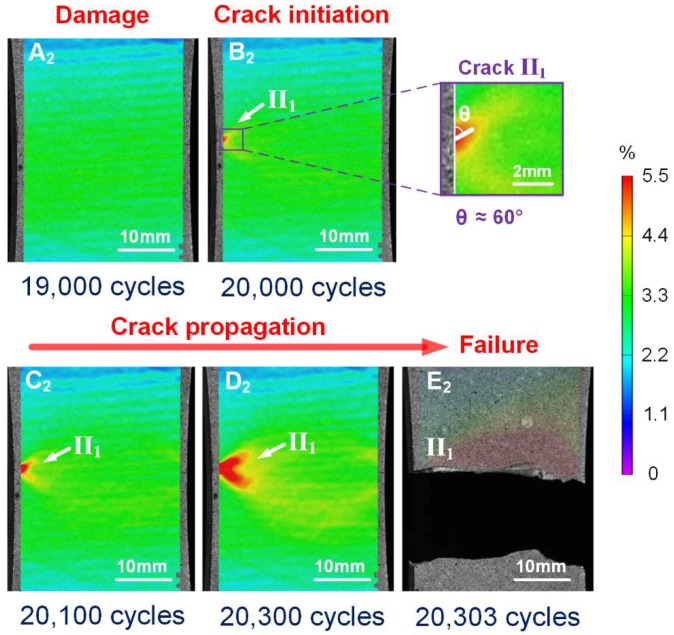
Measured contour-plot distribution of evolution of maximum tensile strain, facilitating visualization of damage, crack initiation and propagation for corrosion time of 48 h.

Figure 8 shows the evolution of maximum tensile strain field in a specimen with a corrosion time of 48 h at zero load level for specified numbers of cycles. Similarly, no evident localized damage is observed before 1.9 × 10^4^ cycles (A_2_ in Figure 8). A strain concentration region II_1_ where the strain value is obviously higher than other regions (B_2_ in Figure 8, indicated by the arrow) appeared at the left edge of specimen after 2 × 10^4^ cycles, indicating crack initiation in this region. The crack was oriented at an angle of $\theta \approx 60^{\circ }$ from the vertical. Compared to the un-corroded specimen, it was also found that the crack initiation region was located near the mid-length of the specimen, but pre-existing localized corrosion damage induced the premature appearance of the crack. The crack quickly grew under fatigue loading from 2 × 10^4^ cycles to 20,300 cycles (B_2_, C_2_ and D_2_ in Figure 8), and led to final failure of the specimen at 20,303 cycles (E_2_ in Figure 8). In contrast to the un-corroded specimen behavior, in this case, just one crack dominated the damage evolution and failure process.

**Figure 9:**
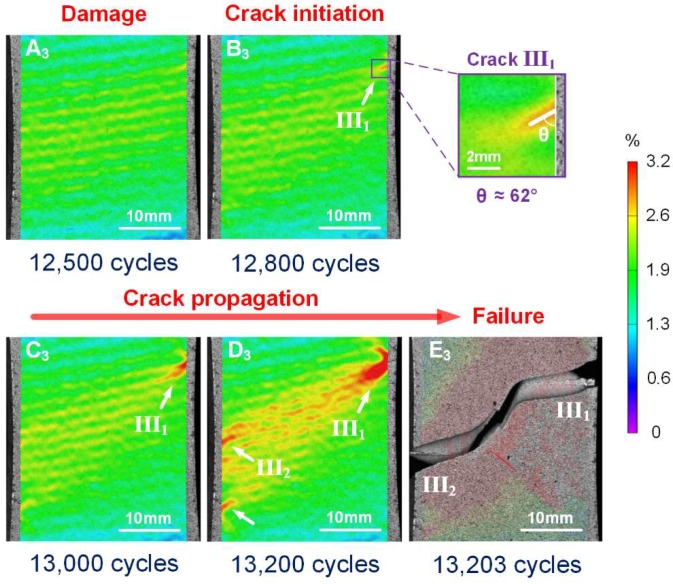
Measured contour-plot distribution of evolution of maximum tensile strain, facilitating visualization of damage, crack initiation and propagation for corrosion time of 96 h.

For the specimen with a corrosion time of 96 h, the damage evolution and fatigue crack propagation can be deduced from the DIC strain maps shown in Figure 9. After damage accumulation and localization, a crack III_1_ nucleated in region III_1_ at the right edge of the specimen after 1.28 × 10^4^ cycles (B_3_ in Figure 9). As shown in the enlarged view of region III_1_, the crack orientation was at the angle of $\theta \approx 62^{\circ }$ from the vertical direction. It was noted that the crack initiation region was away from the specimen mid-length, in contrast to the 0 h and 48 h cases, indicating that corrosion damage has a significant influence on fatigue crack initiation location. The crack gradually enlarged and propagated with increasing fatigue cycles (C_3_ in Figure 9), reflected by the red strain concentration region. It was noted that some other cracks also initiated at the left edge of the specimen (indicated by the white arrows) after 1.32 × 10^4^ fatigue cycles (D_3_ in Figure 9). The quick propagation and coalescence of two main cracks originated from region I_1_ and I_2_ resulted in final failure (E_3_ in Figure 9).

### 3.4. Fracture Morphology Analysis

**Figure 10:**
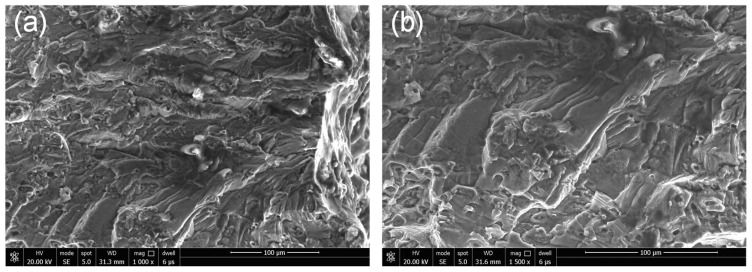
Typical fracture morphology of specimen without corrosion (**a**) crack initiation region, (**b**) cleavage characteristic.

**Figure 11:**
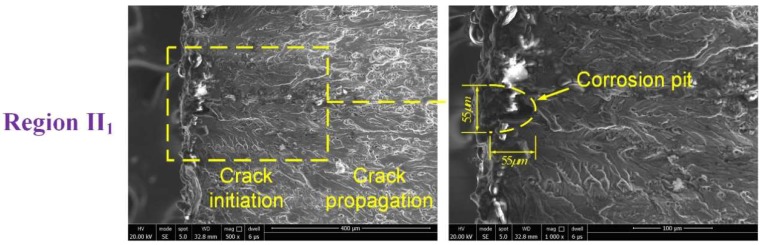
Fracture morphology of specimen with a corrosion time of 48 h.

**Figure 12:**
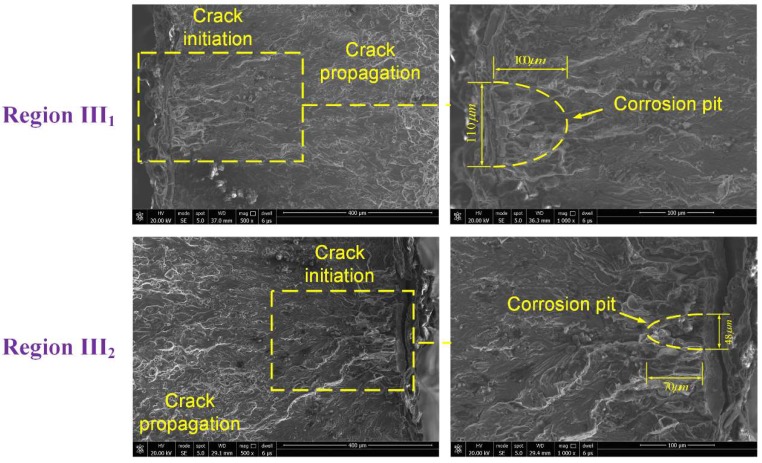
Fracture morphology of specimen with a corrosion time of 96 h.

To further analyze the effect of localized corrosion on fatigue failure, fracture morphology analysis was carried out, focusing on the fatigue crack initiation sites. Figure 10 shows the typical fracture morphology of a failed un-corroded specimen corresponding to Figure 7, indicating a characteristic cleavage nature of the crack initiation morphology. The morphologies of failed 48- and 96-h specimens are shown in Figure 11 and Figure 12, respectively, corresponding to Figure 8 and Figure 9. From the enlarged view of the crack initiation region II_1_ of the 48-h specimen, it was observed that a fatigue crack originated from a corrosion pit penetrated in the specimen edge, which can be approximated as a semi-elliptical shape with width of ~55 μm, and depth of ~55 μm. Similarly, corrosion pits, approximated as semi-elliptical shapes with obviously larger depths, were found in the crack initiation regions III_1_ (width of ~110 μm, depth of ~100 μm) and III_2_ (width of ~48 μm, depth of ~70 μm) for the 96-h specimen. It is deduced from the fatigue-corrosion fracture morphology that crack initiation is caused by the pre-existing localized corrosion. The presence of corrosion pits causing stress concentrations significantly accelerated crack initiation, and the exacerbation by corrosion damage led to the premature crack initiation.

### 3.5. Effect of Localized Corrosion on Damage and Cracking

The significant corrosion-induced degradation in fatigue life and the phenomenon of fatigue crack nucleation from localized corrosion observed here are in agreement with previous reported work [16,20]. In this work, DIC strain maps and fracture morphology, combined with damage evolution curves, facilitate qualitative and quantitative analysis of the temporal-spatial characteristics of damage accumulation, including crack initiation and propagation in the pre-corroded aluminum alloy under fatigue loading. The damage evolution curves reflect the overall fatigue damage accumulation for the whole specimen, while the DIC strain maps give more detailed local characterization of crack initiation and propagation. In addition, the corrosion morphology and sizes of corrosion pits in the key damage regions are identified from the fracture morphology. The experimental results show that prior corrosion causes accelerated damage accumulation, and affects the cycles and location of fatigue crack initiation, as well as the cracking path. Multiple cracks were observed to nucleate at the edge of the specimen, but the primary cracks dominated the failure process. It was found that the location of fatigue crack initiation was determined by the local stress condition and the local corrosion morphology. For example, crack initiation region III_1_, for the most serious corrosion case, was located away from the mid-length of the specimen, as shown in Figure 9 (B_3_). Although the location has a relatively small stress compared to the mid-length, the local corrosion pit here is relatively larger, thus causing localized (microscale) stress concentration. The phenomena demonstrates that corrosion damage can significantly change fatigue crack nucleation location when the initial pre-corrosion damage is severe. The crack orientation from the loading direction, identified by DIC, was affected by prior corrosion. Thus, mixed-mode fatigue cracking behavior needs to be considered. In addition, specific cracks, identified as pre-dominant for the failure process, significantly affect the failure path. For example, in the identified 96-h case, main crack III_1_ was first observed at about 1.28 × 10^4^ cycles (96.9% of fatigue life), as shown in Figure 9 (B_3_), and the final failure was caused by coalescence of cracks III_1_ and III_2_, as shown in Figure 9 (D_3_) and (E_3_). 

## 4. Modelling

### 4.1. Simplified Corrosion-Originated Crack Model

**Figure 13:**
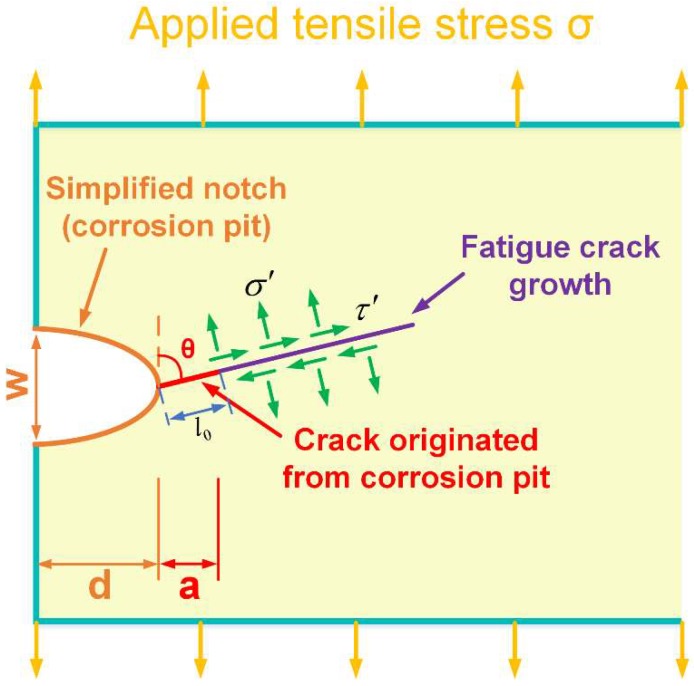
Schematic diagram of simplified mixed-mode crack mode for fatigue crack growth from a corrosion pit.

**Figure 14:**
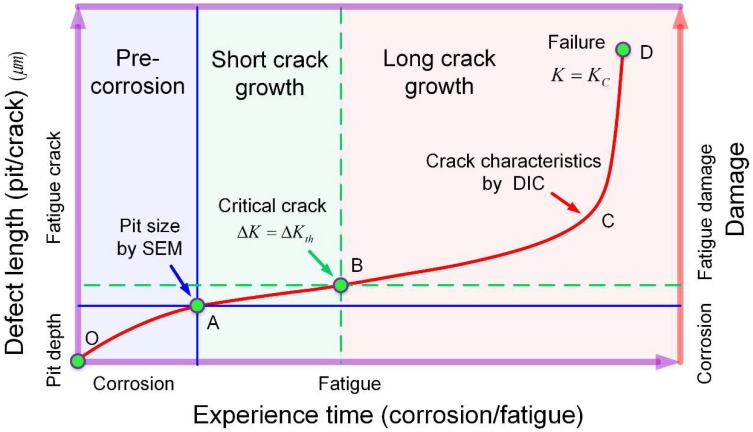
Different stages in pre-corroded fatigue failure process of aluminum alloy.

A single dominated flaw method, considering the behavior of a main crack initiated from the key localized corrosion region, was used to analyze the post-corrosion fatigue failure. Figure 13 shows a schematic of the assumed corrosion-originated, I-II mixed-mode crack. The corrosion pit can be simplified as a semi-ellipse shape notch [26], which grows with increasing corrosion time. The crack is assumed to initiate from the notch root, as denoted by the red arrow, propagating with fatigue cycles (including short crack and long crack growth) and finally leading to failure. It is generally accepted that the transition from short crack to long crack occurs when the stress intensity factor (SIF) range ΔK reaches the threshold SIF range $\Delta K_{th}$, and failure occurs when the SIF K reaches the fracture toughness $K_{C}$ [40,41]. The fatigue failure process of the pre-corroded aluminum alloy can be roughly divided into three stages, namely pre-corrosion, short crack growth, and long crack growth, as shown in Figure 14.

In the pre-corrosion stage, a power-law growth model [42] can be used to describe the increase of corrosion pit depth and width as:

(4) $$d=\eta t^{\kappa }\;$$

(5) $$w=\xi t^{\lambda }\;$$

in which d is the depth of corrosion pit (μm), w is the width of corrosion pit (μm), t is corrosion exposure duration (hour), and η,k,ξ,λ are fitting parameters. In this work, the corrosion depth and width at crack initiation region were measured from the fracture morphology, as shown in Figure 11 and Figure 12. The corrosion depth growth function is identified as $d=1.95t^{0.86}$, and the corrosion width growth function is identified as w=1.15t. 

After that, a crack is assumed to initiate from the root of the corrosion pit, propagating with increasing fatigue cycles until failure, including short crack and long crack growth stages. The critical point for short crack-long crack transition, marked as B in Figure 14, was determined by the threshold SIF range as [40]:

(6) $$\Delta K=\Delta K_{th}\;$$

The failure point, marked as D in Figure 14, was calculated by fracture toughness. Considering the I-II mixed-mode cracking behavior, the fracture criterion can be approximated by the equation of an ellipse [43]:

(7) $${\left(\frac{K_{I}}{K_{IC}}\right)}^{2}+{\left(\frac{K_{II}}{K_{IIC}}\right)}^{2}=1\;$$

where $K_{I}$ is the mode-I SIF, $K_{IC}$ is the mode-I fracture toughness, $K_{II}$ is the mode-II SIF, and $K_{IIC}$ is the mode-II fracture toughness. In this work, $\Delta K_{Ith}\approx 3.9\;MPa\sqrt{m}$ [44], $K_{IC}\approx 32\;MPa\sqrt{m}$ [45], $\Delta K_{IIth}$ and $K_{IIC}$ are assumed to be 10% less than $\Delta K_{Ith}$ and $K_{IC}$ respectively [45].

### 4.2. Fatigue Crack Growth Analysis

For the assumed I-II mixed-mode crack mode, the long crack fatigue growth can be described by a modified Paris crack growth regime [46,47], as shown in Equation 7, which involves the plasticity-induced crack closure effect [48,49].

(8) $$\frac{dl}{dN}=CU^{m}{\left(\Delta K_{eff}\right)}^{m}\;$$

(9) $$\Delta K_{eff}={\left(\Delta K_{I}{}^{2}+\Delta K_{II}{}^{2}\right)}^{\frac{1}{2}}\;$$

(10) U=0.5+0.4R 

where $\frac{dl}{dN}$ is crack growth rate, U is the crack closure coefficient, $\Delta K_{eff}$ is effective stress intensity factor range, C and m are crack growth material parameters and R is the stress ratio.

Short cracks are found to propagate at higher rates than long cracks with an equivalent SIF. EI-Haddad et al. [50] proposed that the growth of a short crack, of length l, can be described by analyzing the crack as if it were actually of length $l+l^{\prime }$, with the EI-Haddad stress intensity factor range $\Delta K_{eff,EH}$ taken as a perturbation of $\Delta K_{eff}$, for short crack growth, as follows:

(11) $$\Delta K_{eff,EH}=\Delta K_{eff}\sqrt{\frac{l+l^{\prime }}{l}}\;$$

where $l^{\prime }$ is referred to as intrinsic crack length, a ‘critical crack length’ from threshold fatigue behavior. Here the $l^{\prime }$ was set as 50 μm [49]. 

**Figure 15:**
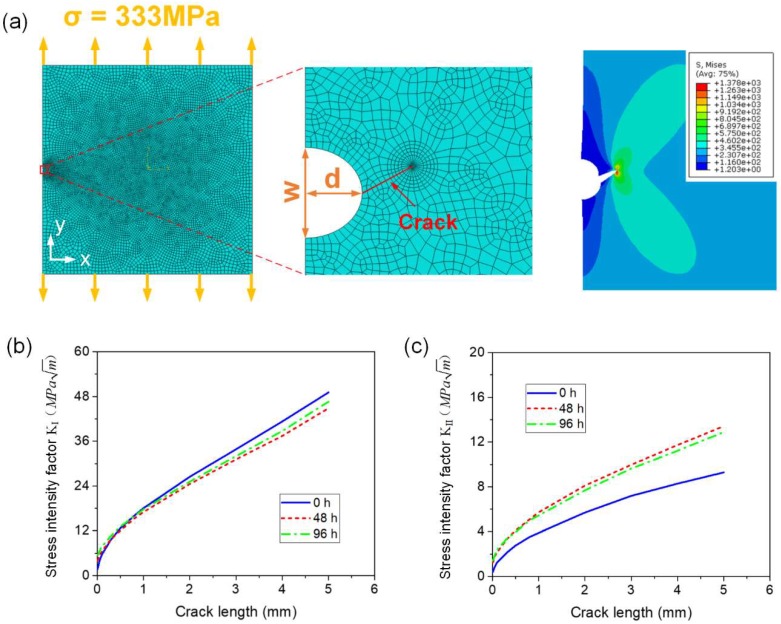
(**a**) Schematic diagram of SIF calculation by Abaqus, (**b**) Calculated Mode-I SIF curves versus crack length for specimens with different corrosion times, (**c**) Calculated Mode-II SIF curves versus crack length for specimens with different corrosion times.

Based on Equations (8) to (11), it is necessary to calculate the Mode-I and Mode-II SIF range versus crack length for fatigue crack growth analysis. To our knowledge, there is no theoretical solution of SIF for the mixed-mode crack shown in Figure 13. Therefore, in this study, the mode-I and mode-II SIFs for specimens with identified corrosion pit sizes and different crack lengths were calculated by the commercial non-linear, finite element software Abaqus. The schematic diagram is shown in Figure 15a. A 2D model corresponding to Figure 13 was built to calculate the SIF with a specified crack length based on contour integral analysis (no crack growth). The geometric properties were set based on the experimental tests. As described above, corrosion pit was simplified as a notch with the identified depths and widths. The inclined crack originating from the root of the notch, with a specified orientation (determined by DIC in Section 3.3), was assigned as a seam in Abaqus. The contour integral crack analysis method was chosen for the SIF calculation. Three-noded, plane stress (CPS3 in Abaqus) elements were used to form the mesh around the crack tip, while four-noded, plane stress (CPS4R) elements were used elsewhere. A very fine mesh was defined around the crack tip for accurate SIF determination, gradually becoming coarser with increasing distance from the crack tip, to ensure the mesh convergence. Linear elastic behavior was assumed with a Young’s modulus of 70 GPa, and a Poisson’s ratio of 0.3. A load of 333 MPa was applied on the top section, to match the experimental fatigue test load. Nodes on the bottom section were constrained in X-direction and Y-direction. Then the Mode-I and Mode-II SIF value for a specified crack length (σ_max_ = 333 MPa) was obtained via Abaqus. Changing the specified crack length, and repeating this process, furnished the curves of Figure 15b,c for Mode-I and Mode-II SIF versus crack length by interpolation.

**Figure 16:**
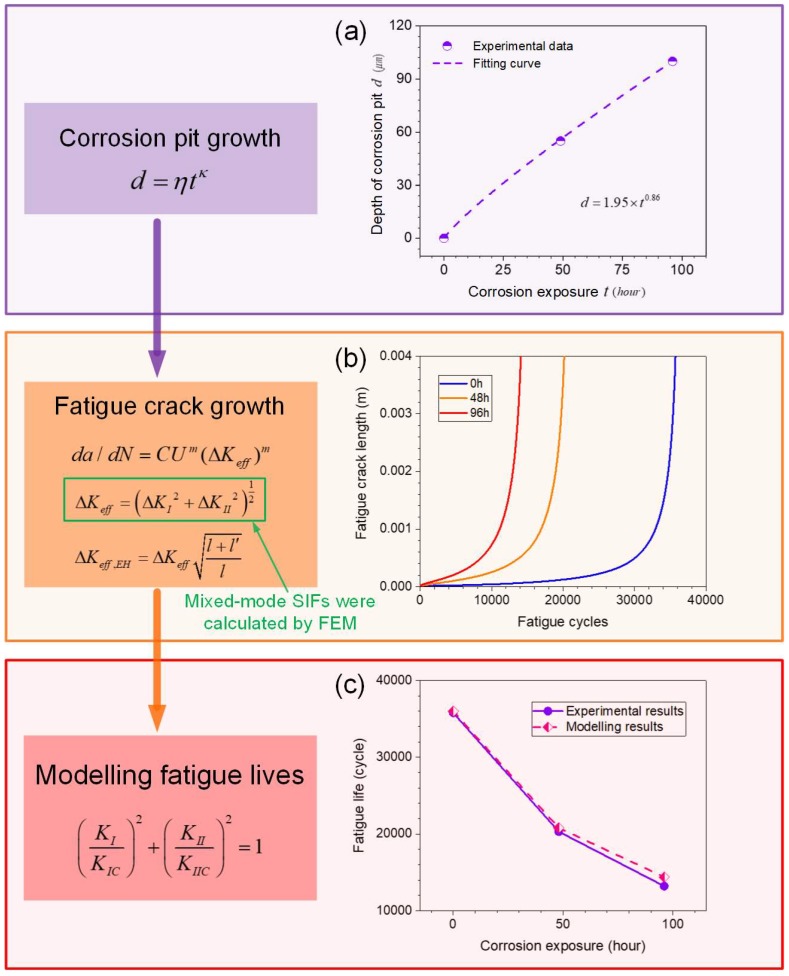
(**a**) Pit depth growth versus corrosion time, (**b**) fatigue crack growth for specimens with different corrosion times, (**c**) predicted and measured fatigue lives of specimens with different corrosion times.

Combining the calculated SIF curves with initial crack lengths and modified Paris’ equation Equations (8) to (11), the fatigue crack propagation lengths in each cycle interval dN can be computed until final fracture, defined by Equation (7). For the un-corroded case, the fatigue crack initiates primarily from micro-structural features, such as inclusion particles, voids or slip-band formation; and typical previously-published values for initial defect size for the 2024 aluminum alloy range between as 6–20 μm [51]. In this paper, the initial crack length for these specimens is assumed to be $l_{0}=10\;\mu m$. The curve of corrosion depth versus corrosion time is shown in Figure 16a. The crack growth material parameters were calibrated for the un-corroded specimen as $C=1.98\times 10^{-10}m/(cycleMPa\sqrt{m})$, m=3.4 and these were also assumed to be applicable to life prediction of pre-corrosion specimens. Although corrosion-fatigue tests of aluminum alloy, performed in corrosion solution, have shown that fatigue crack growth parameters exhibit different values in different environments [25,52], it has been argued that it is acceptable to adopt constant material parameters, independent of environment, in the case of pre-corrosion aluminum alloy, for fatigue life prediction [6,7]. In any case, the rationale here is that the key effect of pre-corrosion is to influence the initial damage. The mechanical parameters and calibrated model coefficients are listed in Table 2. Based on Equations (7) to (11), the fatigue crack growth of specimens with different corrosion times was calculated, as shown in Figure 16b. The fatigue lives was also predicted based on Equation 6, as shown in Figure 16c. It is clear that the predicted fatigue lives are consistent with the experimental results.

It is intended that the increased understanding and post-corrosion fatigue cracking models presented in this paper will contribute directly to the development of methodologies for corrosion management, to ensure increased flight safety and decreased maintenance costs.

## 5. Conclusions

With the combined analysis of damage curves, strain maps by 3D-DIC and fracture morphology by SEM, the damage accumulation, crack initiation and propagation of aluminum alloy 2024-T4 with different levels of initial corrosion damage in constant amplitude fatigue tests were experimentally and numerically investigated. It is concluded that:

(1) The combined analysis of fatigue tests, DIC and SEM is effective to characterize damage evolution and crack propagation in pre-corroded aluminum alloy under fatigue loading conditions.
(2) The fatigue life of test specimens reduced by about 47% and 68% after prior corrosion exposure of 48 h and 96 h, respectively. A nonlinear damage accumulation model was calibrated and used to describe the fatigue damage evolution. It was shown that prior corrosion led to the accelerated damage accumulation with increasing corrosion time.
(3) The DIC results showed that cracks always appeared at the edge of the specimen. Corrosion damage significantly affects the fatigue crack nucleation location when the corrosion damage is serious. Multiple cracks were observed with a notable influence on final fracture path. The fracture morphology clearly shows that the presence of corrosion pits with increased depth causing stress concentrations greatly accelerates crack initiation.
(4) Based on the experimental observations, an approximate mixed-mode crack model, was developed and combined with corrosion pit sizes, to successfully capture the general effect of pre-corrosion of fatigue cracking behavior, based on a single dominated localized corrosion region.

## Figures and Tables

**Table 1:** Identified damage parameters for specimens with different corrosion times.

Corrosion Exposure *T*(*h*)	*α*	*β*	*p*
0	0.004	1.000	2.325
48	0.005	1.001	2.328
96	0.004	1.003	1.943

**Table 2:** Fatigue properties of aluminum alloy 2024-T4.

Parameter	Value
$\Delta K_{Ith}(MPa\sqrt{m})$	3.9 [44]
$K_{IC}(MPa\sqrt{m})$	32 [45]
$\Delta K_{IIth}(MPa\sqrt{m})$	3.5 [44]
$K_{IIC}(MPa\sqrt{m})$	28.8 [45]
$l^{\prime }(\mu m)$	50 [47]
$l_{0}(\mu m)$	10 [49]
$C(m/(cycleMPa\sqrt{m}))$	1.98 × 10^−10^
m	3.4

## References

[1] Hu P., Meng Q.C., Hu W.P., Shen F., Zhan Z.X., Sun L.L. (2016). A continuum damage mechanics approach coupled with an improved pit evolution model for the corrosion fatigue of aluminum alloy. Corros. Sci..

[2] Sankaran K.K., Perez R., Jata K.V. (2001). Effects of pitting corrosion on the fatigue behavior of aluminum alloy 7075-T6: Modeling and experimental studies. Mater. Sci. Eng. A.

[3] Molent L. (2015). Managing airframe fatigue from corrosion pits – A proposal. Eng. Fract. Mech..

[4] Barter S.A., Molent L. (2014). Fatigue cracking from a corrosion pit in an aircraft bulkhead. Eng. Fail. Anal..

[5] Chen Y.J., Liu C.C., Zhou J., Wang X.C. (2017). Multiaxial fatigue behaviors of 2024-T4 aluminum alloy under different corrosion conditions. Int. J. Fatigue..

[6] Huang Y.F., Ye X.B., Hu B.R., Chen L.J. (2016). Equivalent crack size model for pre-corrosion fatigue life prediction of aluminum alloy 7075-T6. Int. J. Fatigue..

[7] McMurtrey M., Bae D., Burns J. (2017). Fracture mechanics modelling of constant and variable amplitude fatigue behaviour of field corroded 7075-T6511 aluminium. Fatigue. Fract. Eng. Mater. Struct..

[8] Weber M., Eason P.D., Ozdes H., Tiryakioglu M. (2017). The effect of surface corrosion damage on the fatigue life of 6061-T6 aluminum alloy extrusions. Mater. Sci. Eng. A.

[9] Medved J.J., Breton M., Irving P.E. (2004). Corrosion pit size distributions and fatigue lives—a study of the EIFS technique for fatigue design in the presence of corrosion. Int. J. Fatigue..

[10] Genel K. (2007). The effect of pitting on the bending fatigue performance of high-strength aluminum alloy. Scr. Mater..

[11] Burns J.T., Boselli J. (2016). Effect of plate thickness on the environmental fatigue crack growth behavior of AA7085-T7451. Int. J. Fatigue..

[12] Burns J.T., Larsen J.M., Gangloff R.P. (2012). Effect of initiation feature on microstructure-scale fatigue crack propagation in Al–Zn–Mg–Cu. Int. J. Fatigue..

[13] Burns J.T., Gupta V.K., Agnew S.R., Gangloff R.P. (2013). Effect of low temperature on fatigue crack formation and microstructure-scale propagation in legacy and modern Al–Zn–Mg–Cu alloys. Int. J. Fatigue..

[14] Russo S., Sharp P.K., Dhamari R., Mills T.B., Hinton B.R.W., Clark G., Shankar K. (2009). The influence of the environment and corrosion on the structural integrity of aircraft materials. Fatigue. Fract. Eng. Mater. Struct..

[15] Kim S., Burns J.T., Gangloff R.P. (2009). Fatigue crack formation and growth from localized corrosion in Al–Zn–Mg–Cu. Eng. Fract. Mech..

[16] van der Walde K., Brockenbrough J.R., Craig B.A., Hillberry B.M. (2005). Multiple fatigue crack growth in pre-corroded 2024-T3 aluminum. Int. J. Fatigue..

[17] van der Walde K., Hillberry B.M. (2007). Initiation and shape development of corrosion-nucleated fatigue cracking. Int. J. Fatigue..

[18] Jones K., Hoeppner D.W. (2006). Prior corrosion and fatigue of 2024-T3 aluminum alloy. Corros. Sci..

[19] Burns J.T., Larsen J.M., Gangloff R.P. (2011). Driving forces for localized corrosion-to-fatigue crack transition in Al–Zn–Mg–Cu. Fatigue. Fract. Eng. Mater. Struct..

[20] Co N.E.C., Burns J.T. (2017). Effects of macro-scale corrosion damage feature on fatigue crack initiation and fatigue behavior. Int. J. Fatigue..

[21] Li X.D., Wang X.S., Ren H.H., Chen Y.L., Mu Z.T. (2012). Effect of prior corrosion state on the fatigue small cracking behaviour of 6151-T6 aluminum alloy. Corros. Sci..

[22] Joshi G., Mall S. (2017). Crack Initiation and Growth from Pre-corroded Pits in Aluminum 7075-T6 Under Laboratory Air and Salt Water Environments. J. Mater. Eng. Perform..

[23] Hu W.P., Shen Q.A., Zhang M., Meng Q.C., Zhang X. (2012). Corrosion-Fatigue Life Prediction for 2024-T62 Aluminum Alloy Using Damage Mechanics-Based Approach. Int. J. Damage. Mech..

[24] Amiri M., Arcari A., Airoldi L., Naderi M., Iyyer N. (2015). A continuum damage mechanics model for pit-to-crack transition in AA2024-T3. Corros. Sci..

[25] Wang C.Q., Xiong J.J., Shenoi R.A., Liu M.D., Liu J.Z. (2016). A modified model to depict corrosion fatigue crack growth behavior for evaluating residual lives of aluminum alloys. Int. J. Fatigue..

[26] Xiang Y., Liu Y. (2010). EIFS-based crack growth fatigue life prediction of pitting-corroded test specimens. Eng. Fract. Mech..

[27] Sutton M.A., Orteu J.J., Schreier H.W. (2009). Image Correlation for Shape, Motion and Deformation Measurements: Basic Concepts, Theory and Applications.

[28] Song H., Zhang H., Fu D., Zhang Q. (2016). Experimental analysis and characterization of damage evolution in rock under cyclic loading. Int. J. Rock. Mech. Min..

[29] Pan B., Qian K., Xie H., Asundi A. (2009). Two-dimensional digital image correlation for in-plane displacement and strain measurement: a review. Meas. Sci. Tech..

[30] Song H., Zhang H., Fu D., Kang Y., Huang G., Cai Z. (2013). Experimental study on damage evolution of rock under uniform and concentrated loading conditions using digital image correlation. Fatigue. Fract. Eng. Mater. Struct..

[31] Jiang R., Pierron F., Octaviani S., Reed P.A.S. (2017). Characterisation of strain localisation processes during fatigue crack initiation and early crack propagation by SEM-DIC in an advanced disc alloy. Mater. Sci. Eng. A.

[32] Mello A.W., Nicolas A., Sangid M.D. (2017). Fatigue strain mapping *via* digital image correlation for Ni-based superalloys: The role of thermal activation on cube slip. Mater. Sci. Eng. A.

[33] Kovac J., Alaux C., Marrow T.J., Govekar E., Legat A. (2010). Correlations of electrochemical noise, acoustic emission and complementary monitoring techniques during intergranular stress-corrosion cracking of austenitic stainless steel. Corros. Sci..

[34] Duff J.A., Marrow T.J. (2013). *In situ* observation of short fatigue crack propagation in oxygenated water at elevated temperature and pressure. Corros. Sci..

[35] Song H., Bai Z., Zhang H., Niu Y., Leen S. (2018). Effect of pre-corrosion on damage evolution and crack propagation in aluminum alloy 7050-T7651. Fatigue. Fract. Eng. Mater. Struct..

[36] (2001). Standard A G34-01: Standard Test Method for Exfoliation Corrosion Susceptibility in 2XXX and 7XXX Series Al Alloys.

[37] Lemaitre J., Chaboche J.L. (1990). Mechanics of Solid Materials.

[38] Xiao J.Q., Ding D.X., Xu G., Jiang F.L. (2009). Inverted S-shaped model for nonlinear fatigue damage of rock. Int. J. Rock. Mech. Min..

[39] De Matos P.F.P., Nowell D. (2009). Experimental and numerical investigation of thickness effects in plasticity-induced fatigue crack closure. Int. J. Fatigue..

[40] Li S.X., Akid R. (2013). Corrosion fatigue life prediction of a steel shaft material in seawater. Eng. Fail. Anal..

[41] Larrosa N., Akid R., Ainsworth R. (2017). Corrosion-fatigue: a review of damage tolerance models. Int. Mater. Rev..

[42] Turnbull A., McCartney L.N., Zhou S. (2006). A model to predict the evolution of pitting corrosion and the pit-to-crack transition incorporating statistically distributed input parameters. Corros. Sci..

[43] Broek D. (2012). Elementary Engineering Fracture Mechanics.

[44] Liu L. (2008). Modeling of mixed-mode fatigue crack propagation. Ph.D. Thesis.

[45] Spear A.D. (2014). Numerical and experimental studies of three-dimensional crack evolution in aluminum alloys: Macroscale to microscale. Ph.D. Thesis.

[46] Elber W. (1971). The significance of fatigue crack closure. Damage Tolerance in Aircraft Structures.

[47] Bogdanov S. (2015). Fatigue Life Prediction Based on the Advanced Fatigue Crack Growth Model and the Monte-Carlo Simulation Method. Ph.D. Thesis.

[48] Newman J. (1981). A crack-closure model for predicting fatigue crack growth under aircraft spectrum loading. Methods and Models for Predicting Fatigue Crack Growth under Random Loading.

[49] Wang Q., Zhang W., Jiang S. (2015). Fatigue life prediction based on crack closure and equivalent initial flaw size. Materials.

[50] El Haddad M.H., Topper T.H., Smith K.N. (1979). Prediction of non propagating cracks. Eng. Fract. Mech..

[51] Newman J.C., Phillips E.P., Swain M.H. (1999). Fatigue-life prediction methodology using small-crack theory. Int. J. Fatigue..

[52] Meng X., Lin Z., Wang F. (2013). Investigation on corrosion fatigue crack growth rate in 7075 aluminum alloy. Mater Design..

